# Insulin-Increased L-Arginine Transport Requires A_2A_ Adenosine Receptors Activation in Human Umbilical Vein Endothelium

**DOI:** 10.1371/journal.pone.0041705

**Published:** 2012-07-23

**Authors:** Enrique Guzmán-Gutiérrez, Francisco Westermeier, Carlos Salomón, Marcelo González, Fabián Pardo, Andrea Leiva, Luis Sobrevia

**Affiliations:** 1 Cellular and Molecular Physiology Laboratory (CMPL), Division of Obstetrics and Gynaecology, Medical Research Centre (CIM), School of Medicine, Faculty of Medicine, Pontificia Universidad Católica de Chile, Santiago, Chile; 2 Vascular Physiology Laboratory, Department of Physiology, Faculty of Biological Sciences, Universidad de Concepción, Concepción, Chile; Virgen Macarena University Hospital, School of Medicine, Spain

## Abstract

Adenosine causes vasodilation of human placenta vasculature by increasing the transport of arginine via cationic amino acid transporters 1 (hCAT-1). This process involves the activation of A_2A_ adenosine receptors (A_2A_AR) in human umbilical vein endothelial cells (HUVECs). Insulin increases hCAT-1 activity and expression in HUVECs, and A_2A_AR stimulation increases insulin sensitivity in subjects with insulin resistance. However, whether A_2A_AR plays a role in insulin-mediated increase in L-arginine transport in HUVECs is unknown. To determine this, we first assayed the kinetics of saturable L-arginine transport (1 minute, 37°C) in the absence or presence of nitrobenzylthioinosine (NBTI, 10 µmol/L, adenosine transport inhibitor) and/or adenosine receptors agonist/antagonists. We also determined hCAT-1 protein and mRNA expression levels (Western blots and quantitative PCR), and *SLC7A1* (for hCAT-1) reporter promoter activity. Insulin and NBTI increased the extracellular adenosine concentration, the maximal velocity for L-arginine transport without altering the apparent *K*_m_ for L-arginine transport, hCAT-1 protein and mRNA expression levels, and *SLC7A1* transcriptional activity. An A2AAR antagonist ZM-241385 blocked these effects. ZM241385 inhibited *SLC7A1* reporter transcriptional activity to the same extent in cells transfected with pGL3-hCAT-1^−1606^ or pGL3-hCAT-1^−650^ constructs in the presence of NBTI + insulin. However, *SLC7A1* reporter activity was increased by NBTI only in cells transfected with pGL3-hCAT-1^−1606^, and the ZM-241385 sensitive fraction of the NBTI response was similar in the absence or in the presence of insulin. Thus, insulin modulation of hCAT-1 expression and activity requires functional A_2A_AR in HUVECs, a mechanism that may be applicable to diseases associated with fetal insulin resistance, such as gestational diabetes.

## Introduction

The endogenous purine nucleoside adenosine causes vasodilation of the human placenta vasculature [1]–[5], via a mechanism involving transport of the cationic amino acid L-arginine [4]–[6]. In mammalian cells, L-arginine transport occurs via the human cationic amino acid transporters (hCAT) family, which includes at least five isoforms: hCAT-1, hCAT-2A, hCAT-2B, hCAT-3 and hCAT-4. Of these isoforms, hCAT-1 and hCAT-2B are functional in human umbilical vein endothelial cells (HUVECs) [4]–[6]. Adenosine increases endothelial L-arginine transport mediated by the high-affinity, low capacity, Na^+^-independent hCAT-1 isoform via activation of A_2A_-adenosine receptors (A_2A_AR) in HUVECs [6]. In subjects with insulin resistance, such as patients with polycystic ovary syndrome [7], A_2A_AR activation results in increased insulin sensitivity. These findings suggest a potential involvement of A_2A_AR on insulin biological effects. In support of this, infusion of adenosine increases insulin sensitivity in patients with type 1 diabetes mellitus (T1DM) [8].

Insulin increases L-arginine transport mediated by hCAT-1, hCAT-1 protein and mRNA expression levels, and *SLC7A1* gene (for hCAT-1) promoter activity in HUVECs [9]. In addition, activation of A_2A_AR is required for human umbilical vein rings relaxation caused by insulin [3]. However, there are no reports regarding the role of A_2A_AR in the modulation of L-arginine transport by insulin in HUVECs [3], [4]. Insulin also reduces adenosine transport via the Na^+^-independent, equilibrative nucleoside transporter 1 (hENT1) in HUVECs [3], [10], a process resulting in accumulation of extracellular adenosine [3]. Altered extracellular adenosine concentration could compromise human fetoplacental endothelium, since its lack of ectonucleosidases needed to reduce adenosine levels [11] results in altered adenosine receptors-mediated biological effects [12].

We hypothesize that A_2A_AR is involved in insulin-mediated increase of L-arginine transport by hCAT-1 in HUVECs. In support of this, an A_2A_AR antagonist (ZM-241385) blocked insulin-stimulated hCAT-1 transport activity and expression. These findings could be crucial in diseases associated with fetal endothelial dysfunction and insulin resistance, such as in gestational diabetes [3], [13] where hCAT-1 expression and activity are increased [3], [4], [14].

## Methods

### Ethics statement

This investigation conforms to the principles outlined in the Declaration of Helsinki, and has received approval from the Ethics Committee of the Faculty of Medicine of the Pontificia Universidad Católica de Chile and the Comisión Nacional de Investigación en Ciencia y Tecnología (CONICYT, Chile). Informed written consent was obtained from all patients.

### Human umbilical cords

Umbilical cords were collected after delivery from full-term normal pregnancies. All pregnancies were single births. The pregnant women included in the study did not smoke or consume drugs or alcohol, and had no intrauterine infection or any other medical or obstetrical complications. Women were normotensive and of normal weight, and exhibited a normal response to the oral glucose tolerance test (OGTT). They were under a normal food regimen during the whole pregnancy period. In addition, newborns (43% female, 57% male) were at term, and of normal birth weight and height (Table 1).

**Table 1:** Clinical characteristics of patients and newborns.

Variables	Normal (n = 21)
*Maternal variables*	
Age (years)	28±2.6 (16–40)
Height (cm)	161±4.5 (156–165)
Weight (kg)	60±2.9 (50–79)
BMI (kg/m^2^)	22±1.4 (19–23)
Systolic blood pressure (mm Hg)	105±3.3 (90–110)
Glycemia basal (mg/dL)	85±3.1 (76–99)
OGTT (mg/dL)	
Glycemia basal	76±4.9 (69–97)
Glycemia 2 hours after glucose	104±3.3 (65–114)
*Newborn variables*	
Sex (female/male)	9/12
Gestational age (weeks)	38±0.9 (37–40)
Birth weight (grams)	3281±16 (2762–3700)
Height (cm)	49±1.2 (36–54)
Ponderal index (grams/cm^3^ ×100)	2.7±0.12 (2.4–2.8)

Data are mean ± SEM (range). OGTT, oral glucose tolerance test; BMI, body mass index.

### Cell culture

HUVECs were isolated by digestion with collagenase (0.25 mg/mL; Collagenase Type II from Clostridium histolyticum (Boehringer, Mannheim, FRG)) of umbilical cord veins obtained at birth from normal pregnancies (n = 21) (37°C, 5% CO2). Cells were cultured up to passage 2 in primary culture medium (PCM: medium 199 (M199, Gibco Life Technologies, Carlsbad, CA, USA), 5 mmol/L D-glucose, 10% new born calf serum, 10% fetal calf serum (FCS) (Gibco), 3.2 mmol/L L-glutamine, 100 U/mL penicillin-streptomycin (Gibco)) [3], [9]. Twenty-four hours prior to experiments, the incubation medium was replaced by M199 containing 2% sera after two rinses with 200 μL in phosphate-buffered saline (PBS) solution ((mmol/L): 130 NaCl, 2.7 KCl, 0.8 Na2HPO4, 1.4 KH2PO4 (pH 7.4, 37°C)).

Experiments were performed in the absence or presence (8 hours) of 1 nmol/L insulin, 30 nmol/L 2-[p-(2-carbonyl-ethyl)-phenylethylamino]-5′-N-ethylcarboxamidoadenosine (CGS-21680 (Sigma, Atlanta, GA, USA), A_2A_AR agonist), 10 nmol/L 4-(2-[7-amino-2-[2-furyl]-[1], [2], [4] triazolo [2,3-a]{1,3,5}triazin-5-yl-amino] ethyl)phenol (ZM-241385 (Sigma), A_2A_AR antagonist), 10 nmol/L 5′-N-ethylcarboxamido adenosine (NECA (Sigma), general adenosine receptor agonist), 30 nmol/L 8-cyclopentyl-1,3-dipropylxanthine (DPCPX (Sigma), A_1_AR antagonist) [13] or 10 μmol/L S-(4-nitrobenzyl)-6-thio-inosine (NBTI (Sigma), inhibitory concentration for hENT1 and hENT2 transport activity) [3].

### Adenosine measurements by high-performance liquid chromatography (HPLC)

Adenosine in the extracellular medium was determined in HUVECs confluent primary cultures as described [3], [6]. In brief, aliquots (200 μL) were mixed with 10 μL of 0.5 mol/L acetate-buffer, 10 μL of 1 μmol/L internal standard (adenosine), and 10 μmol/L of 50% aqueous chloroacetaldehyde. After incubation (80°C, 1 hour) and centrifugation (14000 rpm, 4 minutes), aliquots (80 μL) were injected into an Isco HPLC system (pump model 2350, gradient programmer model 2360, 4.6×250 mm C_18_ reverse-phase column, 5-μm particle size (Chemical Research Data Management System, Lincoln, NE, USA)). Mobile phase was 10 mmol/L citrate-buffer with 4.5% acetonitrile and was run isocratically at 1 mL/minute. Fluorescence detection was achieved at an excitation wavelength of 275 nm and an emission wavelength of 420 nm using a Waters M-470 fluorescence detector. Ratio of the area under the adenosine peaks to the area under the internal standard peak was compared with a standard curve [3], [6].

### L-Arginine transport

Overall 0–1000 μmol/L L-arginine transport (3 μCi/mL L-[^3^H] arginine (NEN, Dreieich, FRG), 1 minute, 37°C) was measured as described [9], [15]. Briefly, transport assays were performed in Krebs ((mmol/L): NaCl 131, KCl 5.6, NaHCO_3_ 25, NaH_2_PO4 1, Hepes 20, CaCl_2_ 2.5, MgCl_2_ 1 (pH 7.4, 37°C)) in cells preincubated (overnight) in PCM containing 2% sera. Cells were exposed (8 hours before transport assays) to PCM 2% sera without or with experimental conditions. Cell viability was assayed by Trypan blue exclusion and was not significantly altered (∼96% of viable cells) by the addition of pharmacological agents used in this study. The cell monolayers were rinsed with ice-cold Krebs to terminate tracer uptake. Radioactivity in formic acid cell digests was determined by liquid scintillation counting, and uptake was corrected for D-(^3^H)mannitol (NEN) disintegrations per minute (d.p.m.) in the extracellular space [9].

Overall L-arginine transport at initial rates (i.e., linear uptake up to 1 minute) was adjusted to the Michaelis-Menten hyperbola plus a nonsaturable, lineal component as previously described [9]. Saturable L-arginine transport kinetic parameters maximal velocity (*V*_max_) and apparent Michaelis-Menten constant (*K*_m_) of transport were calculated as described [9]. The relative contribution of insulin to saturable L-arginine transport kinetic parameters was estimated from the maximal transport capacity (*V*_max_/*K*_m_) values for L-arginine transport by:
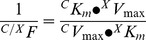
where ^C^ *V*_max_ and ^C^ *K*_m_ are the kinetics parameters for L-arginine transport in control conditions, and ^X^ *V*_max_ and ^X^ *K*_m_ are kinetics parameters of L-arginine transport in HUVECs exposed to different experimental conditions.

### Western blotting

Total protein was obtained from conﬂuent cells washed twice with ice-cold PBS and harvested in 100 μL of lysis buffer (63.7 mmol/L Tris/HCl (pH 6.8), 10% glycerol, 2% sodium dodecylsulphate, 1 mmol/L sodium orthovanadate, 50 mg/mL leupeptin, 5% 2-mercaptoethanol) as described [9]. Cells were sonicated (six cycles, 5 seconds, 100 W, 4°C), and total protein was separated by centrifugation (12000 rpm, 15 minutes, 4°C) as reported [9]. Proteins (50 µg) were separated by polyacrylamide gel (8–10%) electrophoresis and transferred to Immobilon-P polyvinylidene difluoride membranes (BioRad Laboratories, Hertfordshire, UK). The proteins were then probed with primary polyclonal goat *anti*-hCAT-1 (1∶200, 12 hours, 4°C), *anti*-hCAT-2A/B (1∶200, 12 hours, 4°C; against isoforms A (hCAT-2A) and B (hCACT-2B) of hCAT-2) (Santa Cruz Biotechnology, Santa Cruz, CA, USA) and monoclonal mouse *anti*-ß-actin (1∶5000, 1 hour, room temperature) (Sigma Aldrich, St Louis, MO, USA) antibodies. Membranes were rinsed in Tris buffer saline Tween and incubated (1 hour) in TBS-T/0.2% BSA containing secondary horseradish peroxidase-conjugated goat *anti*-goat or *anti*-mouse antibodies (Santa Cruz Biotechnology). Proteins were detected by enhanced chemiluminescence (film exposure time was 2 minutes) and quantified by densitometry as described [9].

### Isolation of total RNA and reverse transcription

Total RNA was isolated using the Qiagen RNAeasy kit (Qiagen, Crawley, UK). RNA quality and integrity were insured by gel visualization and spectrophotometric analysis (OD260/280), quantified at 260 nm. Aliquots (1 μg) of total RNA were reversed transcribed into cDNA as described [3], [9].

### Quantitative RT-PCR

Experiments were performed using a MxPro 3000^TM^ thermal cycler (Stratagene, La Jolla, CA, USA) in a reaction mix containing 0.5 μmol/L primers, and master mix provided in the brilliant SYBR green qPCR Master Mix (Stratagene, La Jolla, CA, USA) as described [3], [9]. SecureStart *Taq* DNA polymerase was activated (15 minutes, 95°C), and the PCR cycling profile included a 95°C denaturation (15 seconds), annealing (20 seconds) at 54°C (hCAT-1 and 28S), and extension (10 seconds) at 72°C (hCAT-1 and 28S). Product melting temperature values were 79.1°C (hCAT-1) and 86.7°C (28S). The oligonucleotide primers used in this study were: hCAT-1 (sense) 5′-GAGTTAGATCCAGCAGACCA-3′, hCAT-1 (*anti*-sense) 5′-TGTTCACAATTAGCCCAGAG-3′, 28S (sense) 5′-TTGAAAATCCGGGGGAGAG-3′, 28S (*anti*-sense) 5′-ACATTGTTCCAACATGCCAG-3′. The number of copies of 28S rRNA was not significantly altered (*P*>0.05, n = 4) in all experimental conditions used in this study (not shown).

### hCAT-1 promoter cloning

Genomic DNA was isolated using the Wizard® SV Genomic DNA Purification System (Promega, Madison, WI, USA). The upstream sequences −1606 and −650 bp from the transcription start codon of *SLC7A1* gene (GenBank: AL596114) were PCR-amplified using Elongase® Enzyme System (Invitrogen) and cloned into pGL3-basic reporter system [9]. The pGL3-hCAT1 reporter constructs generated were pGL3-hCAT1^−1606^ and pGL3-hCAT1^−650^.

### Transient transfection

Sub-confluent (75%) HUVECs primary cultures were resuspended in serum-free M199. Aliquots of cell suspension (0.5 mL, 3.2×10^6^ cells/mL) were mixed with 10 μg of pGL3-hCAT1^−1606^ or pGL3-hCAT1^−650^ reporter constructs, pGL3-Basic (empty pGL3 vector), pGL3-Control (Simian Virus 40 promoter (SV40) pGL3 vector), and the internal transfection control vector pRL-TK expressing Renilla luciferase (Promega) as described [9]. After electroporation (300 Volts, 700 μF, 5–10 milliseconds) (Gene Pulser® II System, BioRad, CA, USA), the cells were cultured (48 hours) in M199 containing 2% FCS. Transfection efficiency was estimated by transfection of the pEGFP-N3 vector (Clontech, Mountain View, CA, USA) and fluorescent cells were counted under an inverted fluorescent microscope (Leica DMIL; Wetzlar, Germany) as described [9].

### Luciferase assay

Electroporated cells were lysed in 200 μL passive lysis buffer (Promega), and *Firefly* and *Renilla* luciferase activity was measured using Dual-Luciferase® Reporter Assay System (Promega) in a Sirius luminometer (Berthold Detection System; Oak Ridge, TN, USA) as described [9].

### Human umbilical vein reactivity

Ring segments of 2–4 mm in length were dissected from human umbilical veins in cold (4°C) PBS solution. Vessel rings were mounted in a myograph (610M Multiwire Myograph System, Danish Myo Technology A/S, Denmark) for isometric force measurements in a Krebs physiological solution ((mmol/L): 118.5 NaCl, 4.7 KCl, 25 NaHCO_3_, 1.2 MgSO_4_, 1.2 KH_2_PO_4_, 2.5 CaCl_2_, 5.5 D-glucose, 300 µmol/L L-arginine, pH 7.4). Vein rings were maintained at 37°C and constantly bubbled with a mixture of 95% O_2_/5% CO_2_. The optimal diameter for each vessel was adjusted through the determination of the maximal active response evoked by 62.5 mmol/L KCl [9]. In some vein rings, the endothelium was removed by gentle abrasion of the intimal surface and successful removal of this cell layer is determined by a reduction in the vasodilatation to 0.1 μmol/L calcitonin gene-related peptide (Peptides International, Inc., KY, USA) (not shown) [16]. Umbilical vessel rings were incubated (37°C) in Krebs in the absence or presence of insulin (0.1–100 nmol/L, 5 minutes) and 10 µmol/L NBTI, 10 nmol/L ZM-241385, 30 nmol/L CGS-21680 and/or 10 nmol/L DPCPX. In some experiments, umbilical vessel rings with or without endothelium were exposed (2 minutes or 8 hours) to CGS-21680 in the absence or presence of ZM-241385 or DPCPX. Changes in isometric tension were recorded using the software LabChart (LabChart 7 for Windows, ADInstruments, Australia) coupled to a PowerLab (PowerLab 8/30 Data Acquisition System, ADIntruments, Australia). The tissue responses are as a percentage of maximal contraction induced by 62.5 mM KCl.

### Statistical analysis

Values are mean ± SEM, where n indicates the number of different cell cultures (3–4 replicates). Comparisons between two and more groups were performed by means of Student's unpaired t-test and analysis of variance (ANOVA), respectively. If the ANOVA demonstrated a significant interaction between variables, post hoc analyses were performed by the multiple-comparison Bonferroni correction test. We used the statistical software GraphPad Instat 3.0b and Graphpad Prism 5.0b (GraphPad Software Inc., San Diego, CA, USA) for data analysis. *P*<0.05 was considered statistically significant.

## Results

### Insulin- and NBTI-mediated increase on L-arginine transport

**Figure 1:**
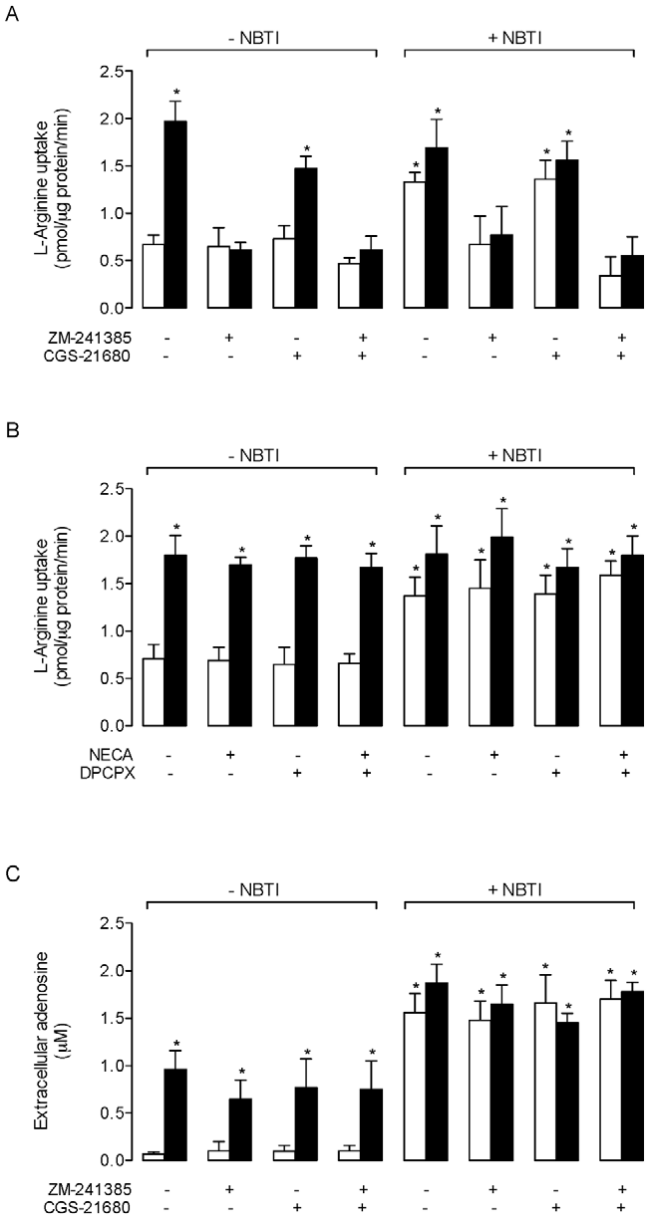
L-Arginine transport and extracellular adenosine concentration. (a) Overall (300 µmol/L) L-arginine transport in HUVECs cultured (8 hours) in absence (white bars) or presence (black bars) of 1 nmol/L insulin, without (–NBTI) or with (+NBTI) 10 µmol/L nitrobenzylthioinosine (NBTI). Cells were co-incubated in culture medium without (–) or with (+) CGS-21680 (30 nmol/L) or ZM-21385 (10 nmol/L). (b) L-Arginine transport in the absence or presence of NECA (10 nmol/L) or DPCPX (10 nmol/L) in cells as in (a). (c) Adenosine concentration in the culture medium in cells as in (a). *P<0.05 versus cells in the absence of NBTI, CGS-21680 and ZM-241385 (control). Values are mean ± SEM (n = 6–18).

L-Arginine transport increased in response to insulin and NBTI. In the absence of NBTI, insulin had no effect on L-arginine transport in cells incubated with ZM-241385 (A_2A_AR antagonist) and ZM-241385 + CGS-21680 (A_2A_AR agonist) (Fig. 1a). Insulin-increased L-arginine transport was not significantly altered by CGS-21680. ZM-241385 or CGS-21680 did not alter basal L-arginine transport in absence of NBTI. Incubation of cells with ZM-241385 or ZM-241385 + CGS-21680, but not CGS-21680 alone blocked the increase of basal L-arginine transport caused by NBTI. Insulin-mediated increase in L-arginine transport was not affected by NBTI. L-Arginine transport increase by insulin or NBTI was not affected by NECA (general ARs agonist) or DPCPX (A_1_AR antagonist) (Fig. 1b).

### Insulin- and NBTI-mediated increase on extracellular adenosine concentration

Extracellular adenosine concentration was increased by insulin and NBTI, an effect not altered by ZM-241385 or CGS-21680 (Fig. 1c). The extracellular level of adenosine was significantly higher in cells exposed to NBTI + insulin than in cells exposed to insulin alone. In addition, NBTI-mediated increase of extracellular adenosine concentration was unaltered in the presence of insulin.

### Insulin- and NBTI-mediated effect on L-arginine transport kinetics

**Figure 2:**
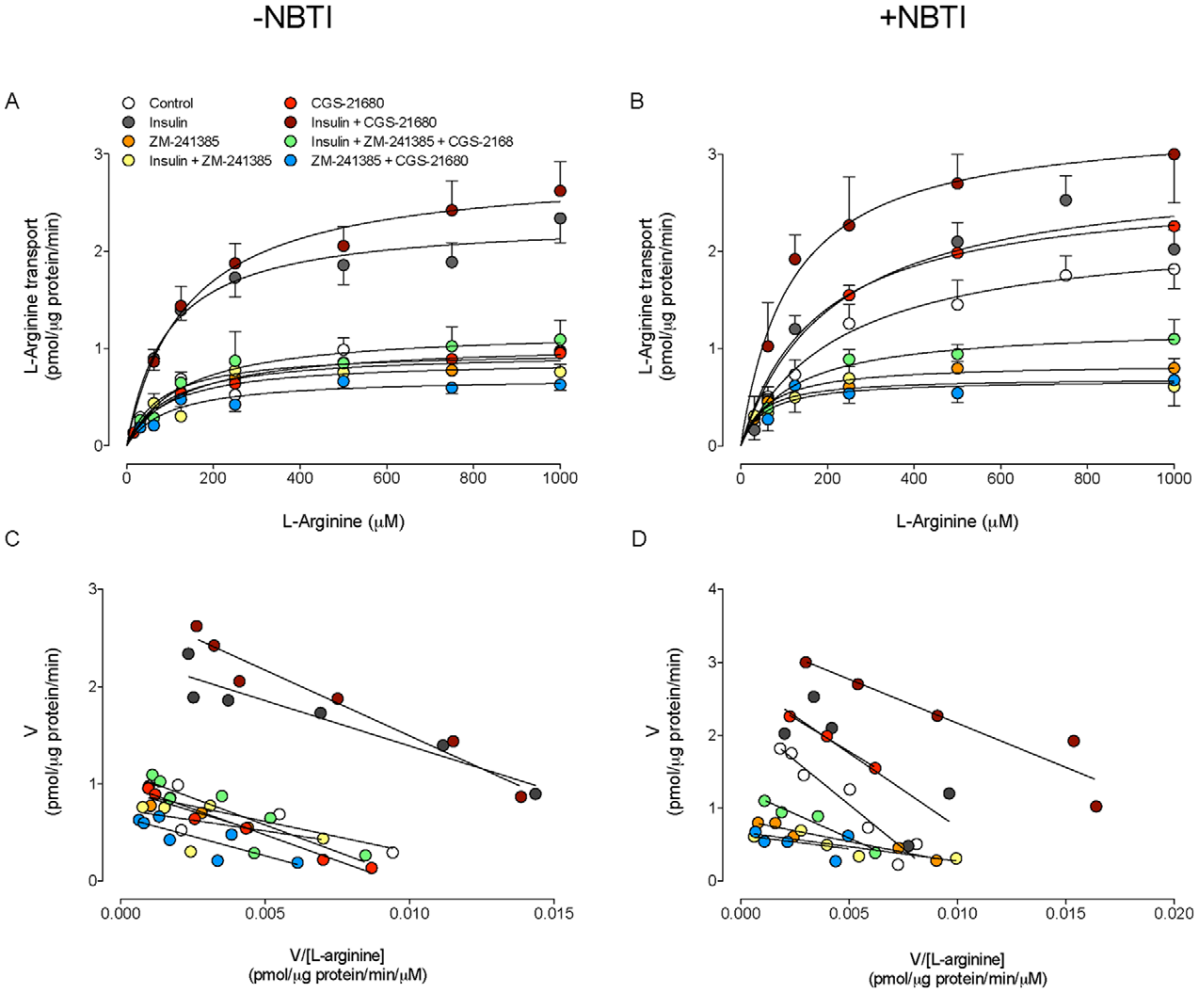
L-Arginine transport kinetics. L-Arginine transport kinetics (3 μCi/mL L-[^3^H] arginine, 1 minute, 37°C) in HUVECs cultured (8 hours) without (–NBTI; a, c) or with (+NBTI; b, d) 10 μmol/L nitrobenzylthioinosine (NBTI). (a, b) Saturable L-arginine transport in absence (control) or presence of 1 nmol/L insulin and/or ZM-241385 (10 nmol/L) or CGS-21680 (30 nmol/L) as indicated. (c, d) Eadie-Hofstee plot of the L-arginine transport data in (a) and (b), respectively. Values are mean ± SEM (n = 6–18).

Overall L-arginine transport was semisaturable in the absence or presence of insulin and/or NBTI, ZM-241385 or CGS-21680 (not shown). Insulin caused an increase in saturable L-arginine transport (Fig. 2a) by increasing the *V*_max_, without altering apparent *K*_m_ (Table 2). This insulin-mediated increase was not affected by CGS-21680, but was abolished by ZM-241385 or ZM-241385 + CGS-21680. However, neither ZM-241385 nor CGS-21685 altered L-arginine transport in the absence of insulin. In cells exposed to NBTI, the saturable L-arginine transport exhibited a higher *V*_max_, without changing the apparent *K*_m_ (Fig. 2b, Table 2). This NBTI-mediated increase in L-arginine transport was unaltered by insulin or CGS-21680, but blocked by ZM-241385 or ZM-241385 + CGS-21680. Similarly, the insulin-mediated increase in L-arginine transport in the presence or absence of NBTI was blocked by ZM-241385 or ZM-241385 + CGS-21680. The Eadie-Hofstee plot of transport data was linear for all experimental conditions in the absence (Fig. 2c) or presence of NBTI (Fig. 2d).

**Table 2:** Kinetic parameters of L-arginine transport.

	L-Arginine transport		
	*V*_max_ (pmol/µg protein/minute)	*K*_m_ (µmol/L)	*V*_max_/*K*_m_ (pmol/µg protein/minute/(µmol/L))
*−NBTI*			
Control	1.0±0.1	79±46	0.0126±0.0085
Insulin	2.3±0.1 *	93±22	0.0215±0.0060*
ZM-241385	1.0±0.1	99±33	0.0101±0.0043
Insulin + ZM-241385	0.9±0.4	95±59	0.0095±0.0101
CGS-21680	1.1±0.2	128±15	0.0086±0.0026
Insulin + CGS-21680	2.9±0.1 *	137±20	0.0212±0.0038 *
CGS-21680 + ZM-241385	0.7±0.1	98±29	0.0071±0.0031
Insulin + CGS-21680 + ZM-241385	1.2±0.1	127±31	0.0094±0.0030
*+NBTI*			
Control	2.2±0.1 *	115±14	0.0195±0.0033 *
Insulin	2.9±0.2 *	161±35	0.0180±0.0052 *
ZM-241385	0.9±0.3	61±12	0.0147±0.0078
Insulin + ZM-241385	0.7±0.4	46±17	0.0152±0.0142
CGS-21680	2.7±0.1 *	151±8	0.0179±0.0016 *
Insulin + CGS-21680	3.4±0.2 *	115±17	0.0296±0.0059 *
CGS-21680 + ZM-241385	1.2±0.1	120±24	0.0100±0.0028
Insulin + CGS-21680 + ZM-241385	0.7±0.1	54±33	0.0129±0.0097

HUVECs were coincubated (8 hours) in the absence (−*NBTI*) or presence (+*NBTI*) of 10 µmol/L nitrobenzylthioinosine (*NBTI*), without (Control) or with insulin (1 nmol/L), ZM-241385 (10 nmol/L) and/or CGS-21680 (30 nmol/L) (see Methods). Maximal velocity (*V*_max_) and apparent Michaelis-Menten constant (*K*_m_) of saturable transport were calculated assuming a single Michaelis-Menten hyperbola. *P<0.05 versus Control in the absence of NBTI. Values are mean ± SEM (n = 19).

### Insulin and NBTI effect on hCAT-1 and hCAT-2A/B expression

**Figure 3:**
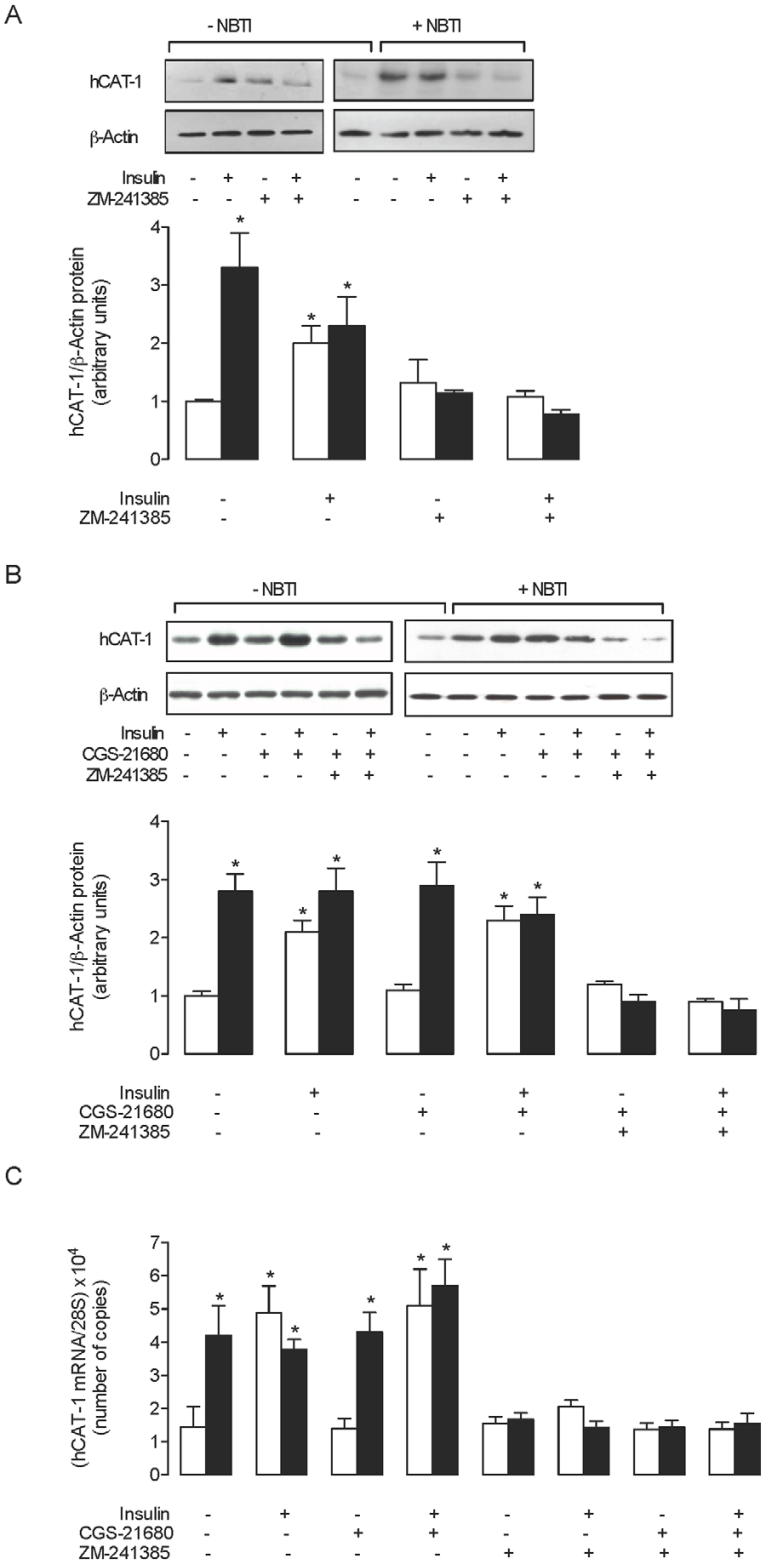
hCAT-1 protein abundance in response to insulin and adenosine receptor agonists and antagonists. (a) Western blots (representative of other 16 experiments) for hCAT-1 in HUVECs incubated (8 hours) without (–NBTI) or with (+NBTI) 10 μmol/L nitrobenzylthioinosine (NBTI), in the absence (–) or presence (+) of 1 nmol/L insulin and/or ZM-241385 (10 nmol/L) (ß-actin is internal control). *Lower panel*: hCAT-1/ß-actin ratio densitometries from data in absence (white bars) or presence (black bars) of NBTI, normalized to 1 in cells in the absence of NBTI, insulin and ZM-241385 (control). (b) Western blots for hCAT-1 in the absence or presence of insulin, CGS-21680 and/or ZM-241385 as in (a). *Lower panel*: hCAT-1/ß-actin ratio densitometries from data in absence (white bars) or presence (black bars) of NBTI normalized to 1 in cells in absence of NBTI, insulin, CGS-21680 and ZM-241385 (control). (c) hCAT-1 mRNA expression relative to 28S rRNA (internal reference) as in (a) and (b). *P<0.05 versus values in control. Values are mean ± SEM (n = 16).

**Figure 4:**
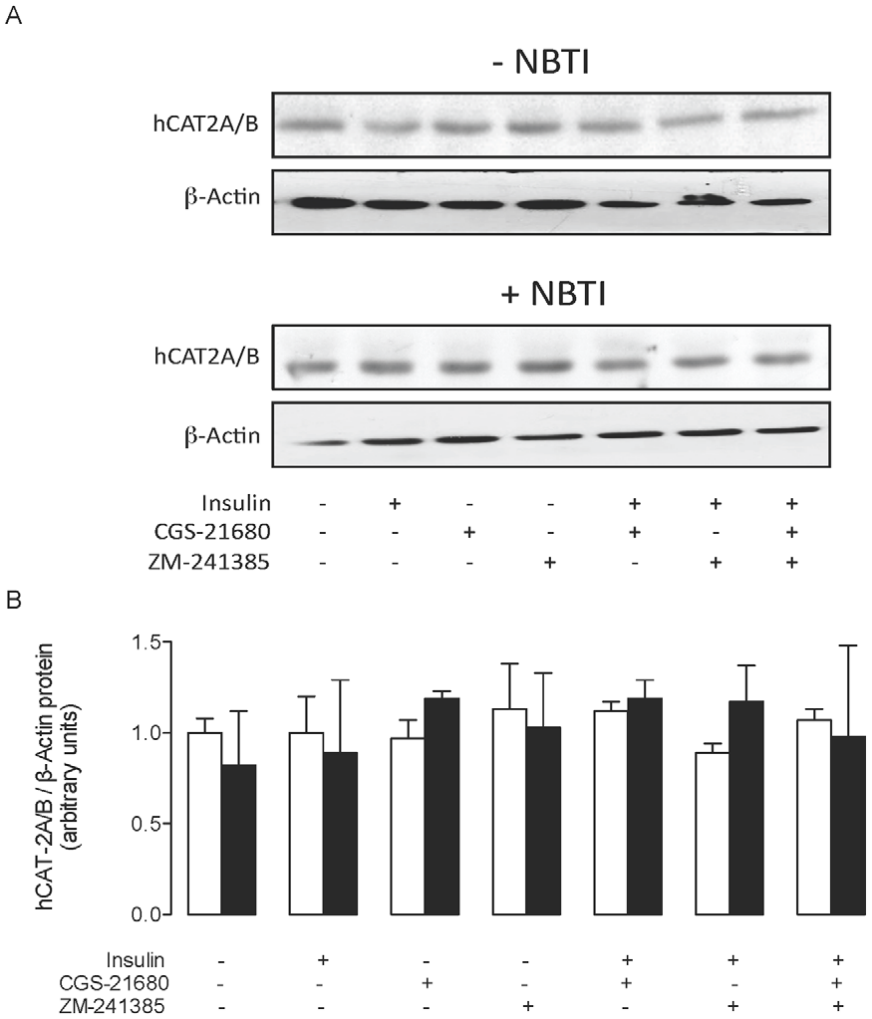
hCAT-2A/B protein abundance in response to insulin and adenosine receptor agonists and antagonists. (a) Western blots (representative of other 6 experiments) for hCAT-2A/B in HUVECs incubated (8 hours) without (–NBTI) or with (+NBTI) 10 μmol/L nitrobenzylthioinosine (NBTI), in the absence (–) or presence (+) of 1 nmol/L insulin, CGS-21680 (30 nmol/L) and/or ZM-241385 (10 nmol/L) (ß-actin is internal control). *Lower panel*: hCAT-2A/B/ß-actin ratio densitometries from data in absence (white bars) or presence (black bars) of NBTI normalized to 1 in cells in the absence of NBTI, insulin, CGS-21680 or ZM-241385 (control). Values are mean ± SEM (n = 6).

The hCAT-1 protein level was higher than control in the presence of insulin or NBTI (Fig. 3a). This insulin-mediated increase on hCAT-1 protein level was not affected by NBTI but abolished by the A_2A_AR antagonist ZM-241385. ZM-241385 blocked the NBTI-mediated increase on hCAT-1 protein level. However, in the absence of insulin or NBTI, ZM-241385 did not have an effect on hCAT-1 protein level. The A_2A_AR agonist CGS-21680 did not alter insulin- or NBTI-mediated increase on hCAT-1 protein level (Fig. 3b) in the absence or presence of ZM-241385. The hCAT-1 mRNA expression levels for all the experimental conditions correlated consistently with the hCAT-1 protein level (Fig. 3c). However, hCAT-2A/B protein level was not significantly altered in any of the experimental conditions used in this study (Fig. 4).

### Transcriptional activity of SLC7A1 promoter

**Figure 5:**
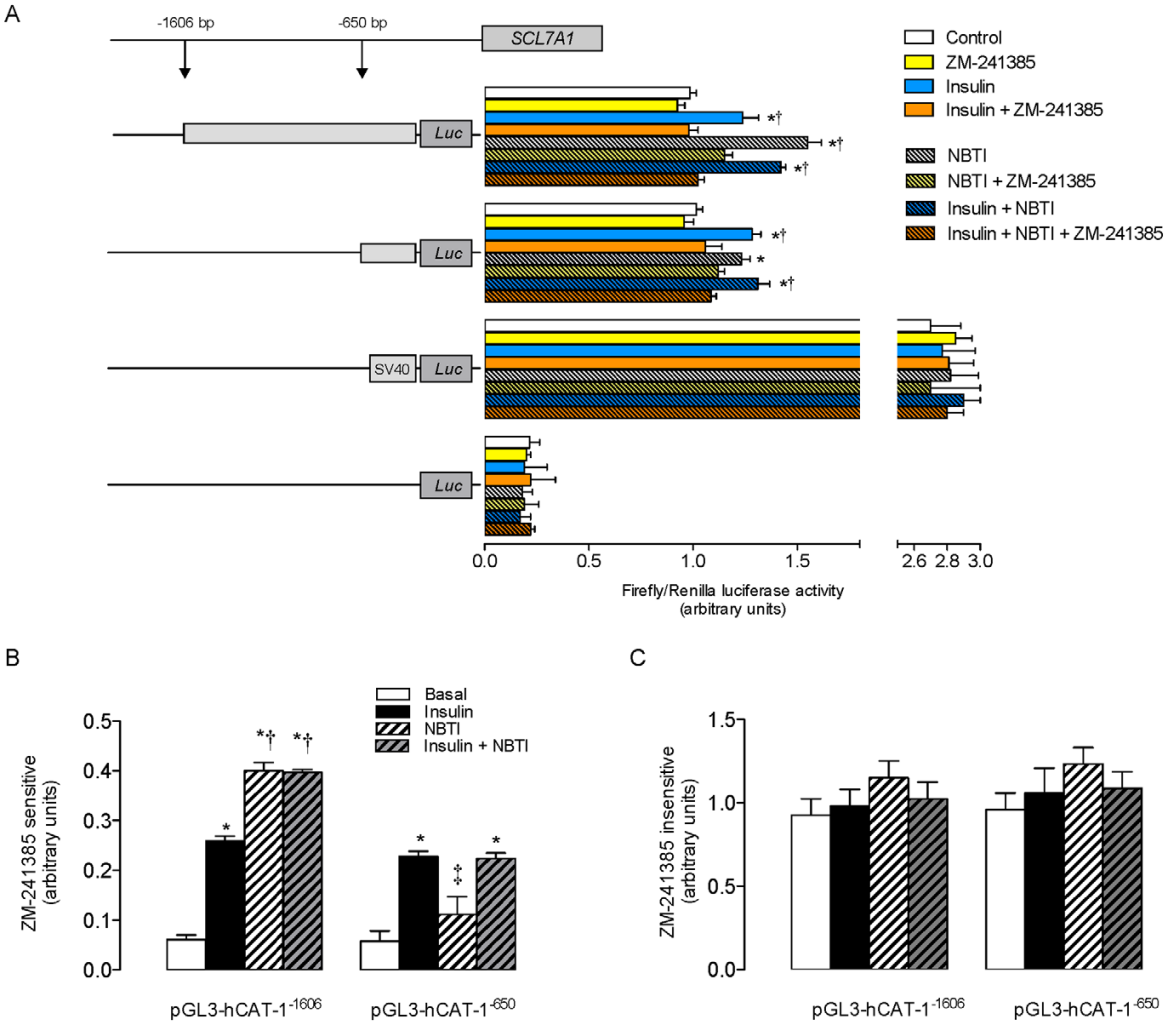
*SLCA71* promoter activity in response to insulin. (a) Luciferase (*Luc*) reporter constructs containing serial truncations of *SLC7A1* promoter (−1606 bp (pGL3-hCAT-1^−1606^) or −650 bp (pGL3-hCAT-1^−1606^) from the transcriptional start point) were transfected in primary cultures of HUVECs incubated (8 hours) without (plain bars) or with (dashed bars) 10 μmol/L nitrobenzylthioinosine (NBTI). Cell transfection was done in the absence or presence of 1 nmol/L insulin and/or ZM-241385 (10 nmol/L), along with *Renilla* reporter plasmid, and assayed for *Firefly* and *Renilla* luciferase activity, respectively. Results depict ratio of *Firefly*/*Renilla* luciferase activity. Cells were also transfected with the empty pGL3-basic vector or pGL3-control vector (SV40 pGL3) as negative or positive controls, respectively. (b) Fraction of *SLC7A1* reporter constructs activity inhibited (sensitive) by ZM-241385 in absence (Basal) or presence of insulin or NBTI as in (a). (c) *SLC7A1* reporter constructs fraction activity non-inhibited (insensitive) by ZM-241385 as in (a). In (a), *P<0.05 versus Control, †*P*<0.05 versus corresponding values in the presence of ZM-241385. In (b), *P<0.05 versus corresponding Basal, †*P*<0.05 versus values in pGL3-hCAT-1^−1606^ in the presence of insulin, ‡*P*<0.05 versus values in pGL3-hCAT-1^−650^ in the presence of insulin or insulin + NBTI. Values are mean ± SEM (n = 6).

*SLC7A1* promoter luciferase activity in the absence of insulin and NBTI (control) was similar in cells transfected with either the pGL3-hCAT-1^−1606^ or pGL3-hCAT-1^−650^ constructs (Fig. 5a). Addition of insulin, NBTI or insulin + NBTI increased promoter activity in cells transfected with either of these constructs. In pGL3-hCAT-1^−1606^ transfected cells, this insulin and NBTI-mediated increase was blocked by ZM-241385. However, in pGL3-hCAT-1^−650^ transfected cells, this A_2A_AR antagonist blocked insulin and (insulin + NBTI)-mediated increase in *SLC7A1* promoter activity. The ZM-241385 sensitive fraction of insulin-mediated increase in *SLC7A1* promoter activity was similar to cells transfected with either constructs, and comparable to the activity in pGL3-hCAT-1^−650^ transfected cells in the presence of NBTI + insulin (Fig. 5b). In addition, ZM-241385 sensitive fraction of NBTI response in pGL3-hCAT-1^−1606^ transfected cells was similar in the absence or presence of insulin. The fraction of the response to insulin or NBTI that was insensitive to ZM-241385 was unaltered in cells transfected with either constructs (Fig. 5c).

### Human umbilical vein reactivity

**Figure 6:**
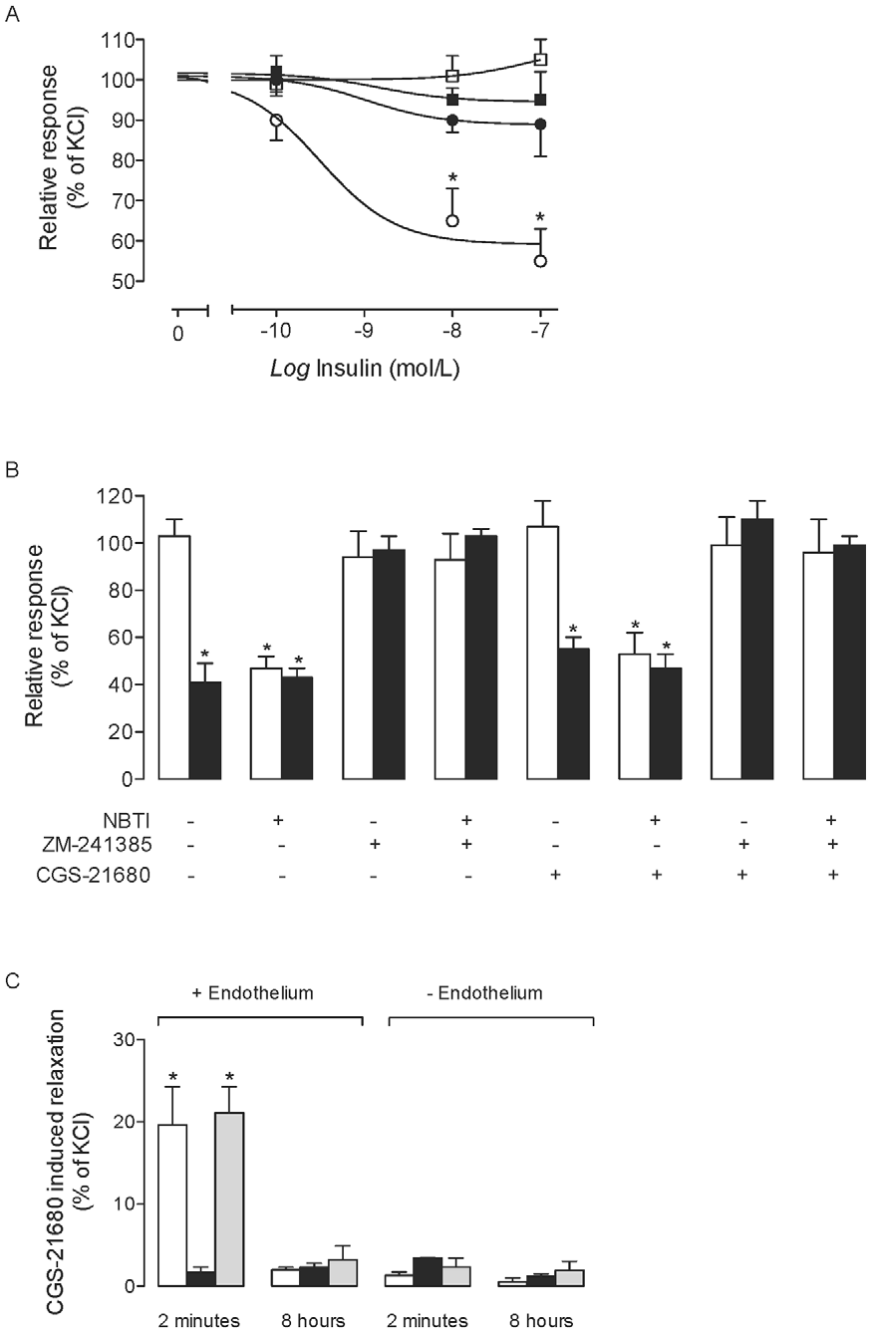
Involvement of adenosine receptors on insulin effect in human umbilical vein rings reactivity. (a) Endothelium-intact (circles) or endothelium-denuded (squares) human umbilical vein rings were exposed (8 hours) to increasing concentrations of insulin in absence (○,□) or presence (•,▪) of ZM-241385 (10 nmol/L). (b) Endothelium-intact human umbilical vein rings incubated in absence (–, control) or presence (+) of 10 μmol/L nitrobenzylthioinosine (NBTI) and/or ZM-241384 as in (a). Reactivity was measured in vessel rings co-incubated without (white bars) or with (black bars) 1 nmol/L insulin. Relative responses are given as a percentage fraction of the initial vessel response to KCl (see Methods). (c) Umbilical vein rings with (+ Endothelium) or without (– Endothelium) were exposed for 2 minutes or 8 hours 30 nmol/L CGS-21680 (white bars) in the absence or presence of 10 nmol/L ZM-241385 (black bars) or 10 nmol/L DPCPX (grey bars). Relative responses are as in (a). In (a), *P<0.05 versus corresponding values in endothelium-intact vessels. In (b), *P<0.05 versus control. In (c), *P<0.05 versus all other values except in DPCPX for 2 minutes in endothelium intact vessels. Values are mean ± SEM (n = 7–9).

Insulin and 1 µmol/L NBTI dilated endothelium-intact human umbilical vein rings (Fig. 6a). ZM-241385 but not CGS-21680 blocked this insulin-mediated dilation in the absence of NBTI (Fig. 6b). However, in the presence of NBTI, these observed changes by insulin and ZM-241385 were unaltered. To test whether CGS-21680 will cause vasodilation via activation of A_2A_AR, umbilical vessel rings were exposed to CGS-21680 for different time periods in the absence or presence of ZM-241385 or DPCPX. Results showed that acute (2 minutes), but not longer periods (8 hours) of incubation with CGS-21680 caused vessel rings vasodilation, an effect blocked by ZM-21680 but unaltered by DPCPX (Fig. 6c). All these molecules were ineffective in endothelium-denuded vessel rings.

**Figure 7:**
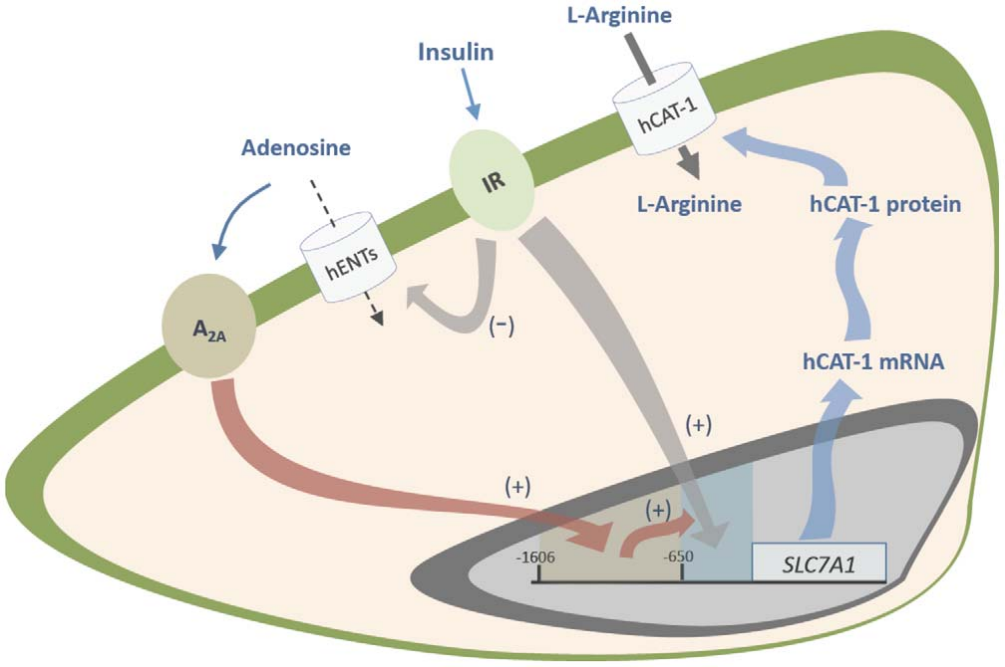
Proposed model for insulin action requiring adenosine receptors on L-arginine transport in human fetal endothelial cells. Human umbilical vein endothelial cells respond to insulin via activation of insulin receptors (IR) leading to a reduced (–) adenosine uptake via the human equilibrative nucleoside transporters (hENTs). Insulin reduced adenosine uptake leading to extracellular accumulation of this nucleoside, which turns into activation of A_2A_ adenosine receptors (A_2A_) at the plasma membrane. Activation of these membrane receptors leads to a mechanism mediated by transcription factors acting as activators (+) between −1606 and −650 bp from the transcription start point of *SLC7A1* (for hCAT-1) gene promoter. Alternatively, insulin activates transcription factors acting as activators (+) between −650 bp and the transcription start point of *SLC7A1*. This phenomenon increases (+) by adenosine receptor-activated transcription factors increasing hCAT-1 mRNA expression and protein abundance resulting in stimulation of L-arginine transport by HUVECs in response to insulin.

## Discussion

This study shows that A_2A_AR activation is required for the insulin-mediated increase in hCAT-1–mediated L-arginine transport in HUVECs. It is also required for insulin-mediated increase in hCAT-1 protein and mRNA expression levels, and *SLC7A1* transcription. Insulin also caused endothelium-dependent dilation of human umbilical vein rings. Activation of A_2A_AR was required only in cells or vein rings challenged with insulin, but not in the absence of this hormone. This observation could result from insulin-mediated increase in extracellular adenosine concentration, likely due to reduced adenosine uptake by the endothelium. These findings suggest that the insulin-mediated effects on human fetal endothelium follow activation of A_2A_AR. It is likely that this phenomenon results from extracellular accumulation of adenosine, secondary to a reduced adenosine uptake. This biological phenomenon could be crucial in diseases where extracellular adenosine is higher than normal, such as in gestational diabetes [3], [13] or in other states of insulin resistance like polycystic ovary syndrome [7] or T1DM [8].

### Insulin-mediated effect on L-arginine transport

L-Arginine transport is mediated by members of the family of human cationic amino acid membrane transporters (hCATs), of which hCAT-1 is the main membrane transporter accounting for L-arginine uptake in HUVECs [15]. hCAT-1 mediated L-arginine transport occurs with an apparent *K*_m_ of ∼150 µmol/L in this cell type. The transport of L-arginine via hCAT-1 is increased by insulin [9], tumour growth factor ß1 (TGF-ß1) [14] and elevated extracellular D-glucose [6], and is reduced in diseases of pregnancy altering fetal growth such as intrauterine growth restriction (IUGR) [15]. Insulin-mediated effects on hCAT-1 activity paralleled changes in hCAT-1 expression, including *SLC7A1* promoter gene transcriptional activity in HUVECs [9]. The results of this study confirm the association between insulin and hCAT-1 maximal transport capacity. Interestingly, the protein levels of hCAT-2A (a high affinity, low capacity hCAT-2 isoform) and hCAT-2B (a high affinity, low capacity hCAT-2 isoform) [17] were not altered by insulin in HUVECs, demonstrating specificity of insulin-mediated effects on hCAT-1.

Insulin-mediated increase on L-arginine transport in HUVECs was dependent on the availability of functional A_2A_AR. This is supported by the observation that insulin stimulatory effect on L-arginine transport was abolished in cells preincubated with 10 nmol/L of ZM-241385. This concentration of ZM-241385 will inhibit A_2A_AR (*K*_i_ ∼1 nmol/L) [18], but not other adenosine receptors such as A_1_AR (*K*_i_ ∼260 nmol/L) [18], A_2B_AR (*K*_i_ ∼30 nmol/L) [19] and A_3_AR (*K*_i_>740 nmol/L) [3], [13], [20] in which a higher ZM-241385 concentration is required for inhibition. Thus, A_2A_AR could be specifically involved in insulin-mediated increase on hCAT-1 expression and activity. Intriguingly, incubation of cells with 30 nmol/L CGS-21680 (a concentration that preferentially activates A_2A_AR (*K*_i_ ∼27 nmol/L)) [3], [20], [21] did not alter L-arginine transport in the absence or presence of insulin. It is known that the time lapse for CGS-21680 occupancy of A_2A_AR at the plasma membrane (i.e., the residence time) is ∼45 minutes [22], and for the closer adenosine derivative 2-chloro-adenosine [12] at these receptors is ∼110 minutes [23]. Thus, the lack of CGS-21680 effect on L-arginine transport could result from A_2A_AR desensitization in response to chronic incubation (8 hours in our study) with this agonist. In addition, the *K*_i_ value for 2-chloro-adenosine (*K*_i_ ∼180 nmol/L) [24] and adenosine (*K*_i_ ∼310 nmol/L) [25] are ∼7 and ∼12 fold, respectively, compared with the reported *K*_i_ for CGS-21680 acting on human A_2A_AR. Thus, we speculate on the possibility that the increase in L-arginine transport observed in cells incubated with NBTI (a condition leading to increased extracellular adenosine), could result from a less pronounced A_2A_AR desensitization caused by adenosine in response to this nucleoside transport inhibitor. Thus, an extended period of incubation of cells with CGS-21680 could result in lack of agonist-A_2A_AR receptor interaction in HUVECs. In fact, we previously reported that 2 minutes incubation with 100 nmol/L CGS-21680 (a concentration that activate A_2A_AR and A_3_AR (*K*_i_ ∼67 nmol/L)) [3], [14], [21] increases L-arginine transport via hCAT-1 in HUVECs [6]. The finding that ZM-241385 blocked the insulin-mediated increase in L-arginine transport in cells incubated with CGS-21680 is paradoxical since, under the experimental conditions used in this study, this agonist could cause A_2A_AR desensitization and reduction in insulin-mediated increase. However, since the affinity of ZM-241385 for these receptors is ∼27 fold higher than CGS-21680 [12], [24], it is likely that insulin-mediated increase was abolished due to a blockage of A_2A_AR by ZM-241385 rather than a desensitization of these receptors by CGS-21680. In addition, 10 nmol/L NECA (a concentration that preferentially activates A_1_AR, *K*_i_ ∼14 nmol/L) [3], [21], [22] and DPCPX (a highly specific A_1_AR antagonist (*K*_i_ ∼4 nmol/L) less effective on A_2A_AR, *K*_i_ ∼130 nmol/L) [3], [22] did not alter the insulin-mediated increase. Since A_1_AR is expressed at minimal levels in HUVECs [26], [27], it is likely that insulin action requires only A_2A_AR in this cell type.

Insulin increases extracellular adenosine concentration in primary cultures of HUVECs [3]. This is likely a consequence of reduced adenosine uptake via human equilibrative nucleoside transporters (hENTs), since NBTI mimicked insulin-mediated increase of extracellular adenosine concentration and did not further alter insulin-increased hCAT-1 expression and activity. It has been shown that adenosine increases hCAT-1–mediated L-arginine transport, and hENT1-mediated adenosine transport in HUVECs via a mechanism involving A_2A_AR activation [2], [5], [28]. Thus, these observations together with our findings suggest that adenosine accumulation in the extracellular medium in cultured HUVECs could be a condition facilitating insulin-mediated effects in this cell type. This phenomenon occurs in human subjects exhibiting insulin resistance, such as in patients with polycystic ovary syndrome. In these patients (R)-N6-phenylisopropyladenosine (PIA, preferential agonist for A_1_AR (*K*_i_ ∼2 nmol/L) and A_3_AR (*K*_i_ ∼33 nmol/L) with less effectiveness on A_2A_AR (*K*_i_ ∼220 nmol/L) and A_3_AR (*K*_i_>150.000 nmol/L)) [3], [20], [29] increased insulin sensitivity accounting for modulation of 2-deoxy-D-glucose uptake in adipocytes [7]. Consistent with this, adenosine infusion in patients with T1DM leads to increased insulin sensitivity and improved myocardial blood flow [9], further supporting a role for adenosine as a modulator of insulin activity.

### Insulin-mediated effect on SLC7A1 expression

Insulin induced an increase in luciferase activity for the reporter constructs pGL3-hCAT1^−1606^ and pGL3-hCAT1^−650^ which confirms the possibility that insulin modulates *SLC7A1* expression by acting on a promoter region between −650 bp and the transcriptional start point (TSP) of hCAT-1 in HUVECs [9]. In addition, NBTI increased the luciferase activity in cells transfected with pGL3-hCAT1^−1606^ but not pGL3-hCAT1^−650^. This may be due to activation of ARs by adenosine, which accumulates in the extracellular space due to NBTI inhibition of hENTs-mediated adenosine transport. Interestingly, ZM-241385 blocked insulin- and NBTI-mediated increase in *SLC7A1* expression suggesting that A_2A_AR activation is necessary for this phenomenon. In addition, the degree of ZM-241385 inhibition of the insulin-mediated increase (i.e., requiring A_2A_AR activation) was similar for both reporter constructs (∼0.25 a.u. luciferase activity). However, the NBTI-mediated effect involving A_2A_AR activation was similar (*P*>0.05) to the insulin-mediated effect for pGL3-hCAT1^−1606^ (∼0.40 a.u.) but significantly lower (∼0.11 a.u., *P*<0.05) than for pGL3-hCAT1^−650^ in response to this hormone. The fraction of NBTI effect inhibited by ZM-241385 in cells transfected with pGL3-hCAT1^−650^ reporter construct was not significantly different from the activity of this promoter fraction in the absence of this inhibitor. Thus, accumulation of extracellular adenosine in response to insulin leads to an increase of *SLC7A1* expression via stimulation of A_2A_AR, which could most likely result in activation of activator transcription factors for consensus sequences between −650 bp and the TSP of this gene [9]. At least two consensus sequences for nuclear factor κB (NFκB) have been described in bovine aortic endothelial cells [30], [31], and four sequences for the general transcription factor specific protein 1 (Sp1) in HUVECs [9] involved in response to insulin, are located between −650 bp and the TSP of *SLC7A1* promoter. Interestingly, the activity of these transcription factors seems associated with increased L-arginine transport in rat alveolar macrophages [32], murine macrophages (RAW264.7 cells) [33] and HUVECs [34]. In addition, Sp1 activity regulation by TGF-ß1 potentially involves A_2A_AR and/or A_2B_AR activation in HUVECs [35]. Thus, it is feasible that Sp1, and perhaps other transcription factors, could be involved in the modulation of *SLC7A1* promoter activity by insulin via activation of these types of adenosine receptors in HUVECs.

On the other hand, since the degree of inhibition by ZM-241385 on NBTI-mediated increase on *SLC7A1* promoter activity was significant only in cells transfected with pGL3-hCAT1^−1606^ construct, it is likely that the resulting increase in extracellular adenosine mediates activation of consensus sequences between −1606 and −650 bp from the TSP of this gene. In addition, since the latter was also found in cells co-incubated with insulin + NBTI, insulin could modulate *SLC7A1* expression at −650 bp from the TSP by a mechanism(s) that requires A_2A_AR-mediated stimulation of transcription factors with consensus sequences between −1606 and −650 bp from the TSP. These transcription factors could either be activators or repressors of insulin-stimulated transcription factors that increase *SLC7A1* expression in HUVECs. This promoter region in HUVECs shows at least four consensus sequences for activated protein-1 (AP1) transcription factor. In rat astrocytes, AP1 increases its activity in response to A_2A_AR stimulation [36]. However, the activity of a single consensus sequence detected for NFκB in HUVECs reduces following A_2A_AR stimulation [37]. Thus, the insulin-mediated increase on *SLC7A1* transcription possibly results from a dynamic interaction between activator and repressor transcriptional factors.

### Insulin-mediated effect on umbilical vein reactivity

Insulin [3], [9], NBTI [34] and adenosine [3] cause dilation of human umbilical vein vessel rings. We have confirmed this, and further showed that the insulin- and NBTI-mediated dilation of umbilical veins is A_2A_AR-dependent as dilation was abolished by ZM-241385. Furthermore, a short (2 minutes), but not longer (8 hours in this study) incubation period of human umbilical vessel rings with CGS-21680 caused dilation that was abolished by ZM-241385. The lack of umbilical vein rings reactivity after long periods of incubation with CGS-21680 could also result from A_2A_AR desensitization. All these findings were dependent on the presence of intact umbilical vein endothelium. Thus, reported vasodilatory effect of insulin on human umbilical vein [3], [9], and perhaps in other human vascular beds where eNOS plays a key role [38], occurs through the umbilical vein endothelium rather than smooth muscle cells.

Our results suggest that hCAT-1 expression and activity are regulated by insulin via a mechanism involving functional A_2A_AR in HUVECs. This may be through a regulation of both adenosine transport and adenosine receptors, a phenomenon also reflected in modulation of human umbilical vein reactivity. Figure 7 depicts a potential mechanism by which this phenomenon occurs. Insulin stimulates insulin receptors leading to increased *SCL7A1* promoter transcriptional activity in which insulin responsive consensus sequences between −650 bp from the TSP of this gene are required. Insulin also reduces adenosine uptake via hENTs in HUVECs, leading to accumulation of adenosine in the extracellular space. These changes result in increased hCAT-1 mRNA expression and protein abundance, leading to stimulation of L-arginine maximal transport capacity via hCAT-1. Since ZM-241385 (an A_2A_AR antagonist) blocks the insulin-mediated increase on hCAT-1 mRNA and protein expression level, a role for A_2A_AR activation in response to insulin is feasible. However, the effects of insulin and adenosine on *SLC7A1* promoter activity were different, suggesting that A_2A_AR involvement is fully required for activation of a fragment between −1606 and −650 bp from the TSP by adenosine, but an A_2A_AR-dependent mechanism is required for insulin effect only on the fragment between −650 bp and the TSP of this gene in HUVECs. The results of this study show for the first time that insulin modulation of L-arginine transport in human fetal endothelium requires activation of these receptors. These findings could be crucial in diseases associated with insulin resistance [7], [8] and insulin resistance-associated diseases of pregnancy where fetal plasma adenosine level is increased [3], such as in gestational diabetes [3], [4], [14]. Based on the findings described in this study, we suggest that an increase in the extracellular adenosine in gestational diabetes [3], a phenomenon also seen in other diseases of pregnancy such as preeclampsia [39], [40], could reflect a potential defence mechanism of the human umbilical vein endothelial cells against insulin resistance. This phenomenon could be crucial to improving vascular dysfunction in the fetoplacental vasculature associated with these pathological conditions [3], [4].
